# Expanding the gelation properties of valine-based 3,5-diaminobenzoate organogelators with *N*-alkylurea functionalities

**DOI:** 10.3762/bjoc.6.114

**Published:** 2010-10-26

**Authors:** Hak-Fun Chow, Chin-Ho Cheng

**Affiliations:** 1 Department of Chemistry, The Chinese University of Hong Kong, Shatin, NT, Hong Kong SAR; 2 The Center of Novel Functional Molecules, The Chinese University of Hong Kong, Shatin, NT, Hong Kong SAR; 3 Institute of Molecular Functional Materials, Areas of Excellence Scheme, University Grants Committee, Hong Kong SAR

**Keywords:** amino acids, hydrophobic effect, organogelators, self-assembly, urea

## Abstract

A new family of valine-containing 3,5-diaminobenzoate derivatives **2** with *N*-alkylurea moieties attached to the valine moieties was prepared. By appending these two new *N*-alkylurea chains to the molecular structure, their organogelating properties were extended from only aromatic solvents, to a wide range of other types of solvents such as alicyclic hydrocarbons, alcohols and polar solvents such as DMSO and DMF. It was also found that a longer *N*-alkylurea chain conferred improved gelation power and higher thermal stability as compared to those of the shorter ones.

## Introduction

Organic gelators are an interesting group of molecules that are able to form a non-covalent three dimensional network with a particular solvent system. The ultimate result is the immobilization of solvent molecules. These molecules possess many potential applications in biomedical science, environmental and separation technology [1–6].

One key problem in the design and synthesis of organogelators lies in the difficulty in predicting their gelation properties beforehand. Most often a subtle change of the substituents of the organgelator can lead to substantial modification in its gelating properties. This is because gelation is the result of a delicate balance of various driving forces. If the binding interactions between the organogelator molecules are too strong, precipitation or crystallization will take place. On the other hand, if the interactions are too weak, dissolution of the organogelator will result. In addition, non-specific interactions such as hydrophobic and van der Waals interactions are difficult to quantify, yet they can play very important roles on a cumulative scale when such interactions actually involve a large ensemble of solvent and gelator molecules.

**Figure 1:**
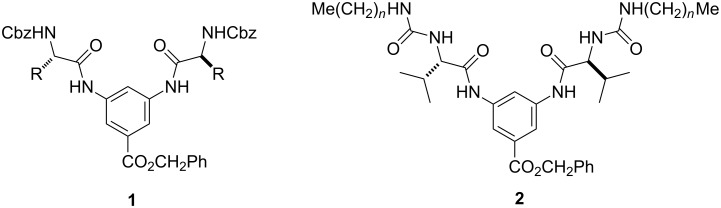
Amino acid based organogelators **1** and **2**.

Our group has been interested in the synthesis and self-assembled gelating properties of amino acid-containing non-dendritic [7–8] and dendritic molecules [9–10]. We recently showed that the organogelation strength in aromatic solvents of a series of α-amino acid-based low molecular weight organogelators **1** (Figure 1) could be enhanced by appending additional aromatic-containing substituents [7]. Both the Cbz protecting group and the benzyl ester functionality were responsible for the enhanced gelating power in aromatic solvents. However, they are poor organogelators in non-aromatic solvents such as alkanes, alcohols, acetone, acetonitrile and DMSO. In order to further expand their gelating power in other solvents, we decided to further modify the appending Cbz groups with other functionalities. One particular interesting moiety is the urea group, which can act simultaneously as a donor and an acceptor of hydrogen bonds and is known to be a key structural element in many organogelators [11–14]. Herein we report that the organogelating properties of **1** could be expanded to include alicyclic hydrocarbon, alcohols and even polar aprotic solvents such as DMSO and DMF by replacing the Cbz group with an *N*-alkylurea functionality (e.g., **2**). In addition, the length of the alkyl chain –(CH_2_)*_n_*Me also exhibited some interesting effects on their gelation ability [15].

## Results and Discussion

### Synthesis

In our earlier report it was shown that, amongst the many α-amino acids used, valine-based **1** (R = CHMe_2_) possessed much better organogelating properties. Hence in the present study we focused only on valine derivatives **2**. A homologous series of *N*-alkylurea side chain derivatives **2** (*n* = 3–6, 9, 10, 12, 15, 18 and 20) was prepared in order to evaluate effect of the length of the alkyl side chain on the resulting gelation properties.

**Scheme 1:**
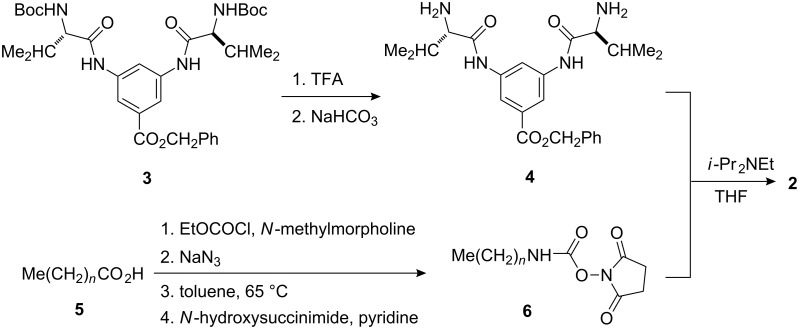
Synthesis of organogelators **2**.

The target organogelators were prepared according to Scheme 1. The known Boc protected benzyl ester **3** [7] was converted into the corresponding diamino compound **4** in 98% yield by treatment with trifluoroacetic acid (TFA) followed by neutralization with NaHCO_3_. The *N*-alkylurea derivatives **2** were then obtained in 88–94% as white solids by coupling compound **4** with various *O*-succinimidyl alkylcarbamates **6**, prepared from the corresponding alkanoic acids **5** according to a literature procedure [16], in the presence of *N*-diisopropylethylamine.

### Structural characterization

All synthesized compounds were characterized by ^1^H and ^13^C nuclear magnetic resonance spectroscopy, mass spectrometry, elemental analysis, and optical polarimetry. Due to the poor solubilities of the target organogelators **2** in chloroform and acetone, their ^1^H and ^13^C NMR spectra were recorded in DMSO-*d*_6_. In addition, the ^1^H and ^13^C NMR spectra of analogues with thirteen or more carbon atoms in the aliphatic hydrocarbon chains were recorded at 100 °C to avoid gelation of the solvent. The ^1^H NMR spectra of compounds **2** showed the presence of the aliphatic hydrocarbon chains and the urea moieties. The methyl group of the aliphatic chains appeared as a triplet at ~ δ 0.86, while the methylenes of the aliphatic chains were located in close proximity to the signals of the valine side chain. On the other hand, the signals from the isopropyl group of the valine residue overlapped with those of the methylenes of the aliphatic chains. The two NH protons of the urea groups were different and appeared as a doublet at δ 6.04 and a triplet at δ 6.07. The benzyl ester group appeared as a singlet at δ 5.24 and a set of multiplets at δ 7.35–7.47. Finally, the aromatic protons of the 3,5-diaminobenzoate moiety appeared as two singlets at δ 7.98 and δ 8.26.

The ^13^C NMR signals of the target molecules **2** could be grouped into three separate regions. First, in the downfield region, the ^13^C signals located at δ 157, 165 and 171 were attributed to the urea, ester and anilide C=O groups, respectively. Second, the ^13^C signals of the central 3,5-diamino-substituted aromatic core were scattered between δ 110–140, while the aromatic signals due to the benzyl ester were found at ~ δ 127–135. Finally, the ^13^C signals of the valine side chain and the aliphatic hydrocarbon side chain were located in a range of δ 12–66. Specifically, the ^13^C signal situated at δ 58 corresponded to the valine α-carbon. The benzylic carbon appeared at δ 66. The remaining aliphatic ^13^C signals due to the aliphatic carbons and some of the resonance peaks overlapped with each other. Hence, the number of the observed signals was sometimes less than theoretically predicted. One ^13^C signal (CH_2_NH) was located at δ 39 and was often buried within the residual solvent signals of DMSO-*d*_6_. For organogelators with longer aliphatic chains, this signal was too weak to be observed and assignment could only be made based on the chemical shift value of other homologues where this signal could be observed.

High-resolution mass spectra were recorded for all synthesized compounds. The experimental determined M^+^ results were in accordance with the theoretical values. Generally, the abundance of the M^+^ decreased with increasing length of the alkyl hydrocarbon side chain.

### Gelation properties

The gelation behavior of bis(*N*-alkylurea valine) benzyl esters **2** were examined at a concentration of 2% w/v in various solvents (Table 1). Most organogelators **2** were found to be insoluble in common non-aromatic organic solvents at room temperature. In contrast, they were soluble in aromatic solvents and organic solvents of high polarity such as 1,4-dioxane, DMF and DMSO upon warming. Interestingly, homologues having shorter aliphatic chain (*n* < 10) were poor organogelators, while the longer aliphatic chain analogues (*n* ≥ 10) formed transparent gels in aromatic solvents and translucent gels in 1,4-dioxane, DMF and DMSO. Most interestingly, it was observed that organogelator **2** (*n* = 20) with the longest hydrocarbon chain also formed opaque gels in alcoholic solvents and translucent gels in alicyclic hydrocarbon solvents, respectively. Hence, the range of the solvents that these new compounds can gel was significantly expanded by replacing the Cbz group with an *N*-alkylurea functionality.

**Table 1:** Gelation behavior of bis(*N*-alkylurea valine) compounds **2** at 2% w/v ^a^.

Solvent	**2** (*n* = 3–6)	**2** (*n* = 9)	**2** (*n* = 10)	**2** (*n* = 12)	**2** (*n* = 15)	**2** (*n* = 18)	**2** (*n* = 20)

THF	I	P	P	P	P	P	P
acetone	I	I	I	I	I	I	I
chloroform	I	I	I	I	I	I	I
dichloromethane	I	I	I	I	I	I	I
ethyl acetate	I	I	I	I	I	I	I
ethylene glycol	I	I	I	I	I	I	I
1-butanol	–	–	–	–	–	–	OG
1-heptanol	–	–	–	–	–	–	OG
1-dodecanol	–	–	–	–	–	–	OG
cyclooctanol	–	–	–	–	–	–	TG
hexane	I	I	I	I	I	I	I
cyclohexene	–	–	–	–	–	–	TG
cyclooctene	–	–	–	–	–	–	TG
1,4-dioxane	S	S	S	TG	TG	TG	TG
DMF	S	S	S	TG	TG	TG	TG
DMSO	S	S	TG	TG	TG	TG	TG
anisole	S	S	CG	CG	CG	CG	CG
benzene	S	S	S	CG	CG	CG	CG
benzyl alcohol	S	CG	CG	CG	CG	CG	CG
*o*-dichlorobenzene	S	CG	CG	CG	CG	CG	CG
nitrobenzene	S	S	TG	TG	TG	TG	TG
toluene	S	CG	CG	CG	CG	CG	CG
*o*-xylene	S	CG	CG	CG	CG	CG	CG

^a^I = insoluble; P = precipitation; S = soluble; CG = clear transparent gel; OG = opaque gel; TG = translucent gel.

Based on these experiments, organogelators **2** with longer aliphatic hydrocarbon chains (*n* ≥ 10) were found to exhibit better gelation behavior in aromatic solvents. However, it was interesting to note that compounds with less than or equal to eight carbon atoms in the side chain failed to induce gelation. The effect of aliphatic hydrocarbon chain length (*n* = 10, 12, 15, 18 and 20) on their gelation power was then compared by the determination of their minimum gelation concentration (MGC) and gel-sol transition temperature (*T*_g_) in different solvents (Table 2). It was found that the longer the alkyl chain, the lower the MGC but the higher the *T*_g_ value. Hence, the cumulative hydrophobic interaction between organogelator molecules with longer aliphatic chains must be stronger than that of the shorter ones, and this factor should contribute to the higher thermal stability of the longer chain organogels. In addition, the presence of the long aliphatic chain also prevented the organogelators from crystallizing by imposing a higher degree of local disorder.

**Table 2:** Minimum gelation concentration and gel-to-sol transition temperature of compounds **2** (*n* = 10, 12, 15, 18 and 20).

Solvent	**2** (*n* = 10)		**2** (*n* = 12)		**2** (*n* = 15)		**2** (*n* = 18)		**2** (*n* = 20)	
	MGC^a^	*T*_g_^b^	MGC	*T*_g_	MGC	*T*_g_	MGC	*T*_g_	MGC	*T*_g_

benzyl alcohol	1.8	—	1.8	70	1.5	79	1.2	88	1.0	94
*o*-dichlorobenzene	1.5	—	1.2	109	1.3	113	1.0	116	0.8	120
*o*-xylene	1.6	—	1.5	75	1.5	80	1.0	83	1.0	86

^a^In w/v%; ^b^In °C as determined by inverted tube method.

Fourier transform infrared spectroscopy (FT-IR) was employed to elucidate the extent of intermolecular hydrogen bonding in the different macroscopic phases of organogelator **2** (*n* = 20) in *o*-xylene (Table 3). In 1% w/v hot *o*-xylene solution (100 °C), two peaks at 3342 and 3247 cm^−1^ in the ν_N–H_ regions were identified. These two signals could be assigned to the stretching bands of urea N–H and anilide N–H, respectively. The two peaks at 1729 and 1629 cm^−1^ in the ν_C=O_ region were found and they were attributed to the anilide C=O and urea C=O, respectively. Signal assignments of the urea C=O and N–H were based on the spectral data of a model compound, namely 1,3-didodecylurea. In the FT-IR spectrum of **2** (*n* = 20) (1% w/v) in *o*-xylene gel at 25 °C, the corresponding peaks were identified at 3335, 3268, 1728 and 1628 cm^−1^, respectively. Hence, there was little difference in terms of the stretching frequencies both in solution and in the gel state. Furthermore, both sets of values are very similar to those of **2** (*n* = 20) in solid KBr, where the C=O and N–H moieties are known to form extensive intermolecular hydrogen bonds. Hence, it is very likely that compound **2** exists as aggregates via intermolecular hydrogen bonding in solution state due to the extremely strong hydrogen bonding property of the urea moiety. In addition, broadening of absorption signals in the gel state was observed which suggests that further intermolecular hydrogen bonding occurred during gel formation.

**Table 3:** FT-IR data of compound **2** (*n* = 20) and 1,3-di(dodecyl)urea^a^.

Samples	urea N–H	anilide N–H	anilide C=O	urea C=O

**2** (*n* = 20) in *o*-xylene solution at 100 °C	3342	3247	1729	1629
**2** (*n* = 20) in *o*-xylene gel at 25 °C	3335	3268	1728	1628
**2** (*n* = 20) in solid in KBr pellet	3348	3248	1730	1629
1,3-di(dodecyl)urea in solid KBr pellet	3340	—	―	1622

^a^in cm^−1^.

## Conclusion

We have reported the synthesis of novel valine-containing 3,5-diaminobenzoate derivatives **2** with additional *N*-alkylurea functionality at the *N*-terminal of the valine residues. The resulting organogelators were found to possess gelating ability that covered a wider range of organic solvents, including alcoholic, aromatic, alicyclic hydrocarbon and polar solvents. Furthermore, attachment of longer aliphatic chains not only enhanced hydrophobic interactions between the organogelators, but also prevented crystallization of the self assembled aggregates during gelation, producing gels with a higher *T*_g_ and a lower MGC value.

## Experimental

**General.** Optical rotation measurements were conducted in DMSO (unless otherwise stated) and below the MGC to avoid molecular aggregation or gelation and were measured with a Perkin Elmer 341 polarimeter. The starting materials **6** were prepared according to a literature procedure [16]. General procedures, yields and characterization data of compounds **6** (*n* = 3–20) can be found in Supporting Information File 1.

**Compound 4.** Trifluoroacetic acid (50 mL, 60 mmol) was added to a solution of the benzyl ester **3** [2] (6.40 g, 10.0 mol) in CH_2_Cl_2_ (100 mL) at 25 °C. The progress of deprotection was monitored by TLC. Upon complete deprotection (~ 12 h), the solvent was removed on a rotary evaporator. The crude product was made alkaline by the addition of aqueous NaHCO_3_ solution to pH 8. The mixture was extracted with CH_2_Cl_2_ (50 mL × 3), the combined organic layers were washed with saturated NaCl solution (100 mL × 2), dried (MgSO_4_), filtered and concentrated in vacuo to give compound **4** as a pale yellow liquid (4.3 g, 98%). [α]_D_^20^ −177.6 (*c* 0.50, CHCl_3_). ^1^H NMR (DMSO-*d*_6_): δ 0.85 (6H, d, *J* = 6.9, CH(C*H*_3_)Me), 0.91 (6H, d, *J* = 6.9, CH(Me)C*H*_3_), 1.85–1.99 (2H, m, C*H*(CH_3_)_2_), 3.12 (2H, d, *J* = 5.7, H_2_NC*H*CH), 3.2–3.5 (6H, brs, NH), 5.35 (2H, s, PhC*H*_2_), 7.36–7.49 (5H, m, Ar*H*), 7.98 (2H, d, *J* = 1.8, Ar*H*), 8.35 (1H, t, *J* = 1.8, Ar*H*). ^13^C NMR (DMSO-*d*_6_): δ 17.4, 19.5, 31.8, 60.8, 66.4, 114.4, 114.7, 128.2, 128.4, 128.6, 130.3, 136.1, 139.6, 165.4, 174.1. MS (FAB): 441 (M + H^+^, 30%). HRMS (LSIMS): calcd for C_24_H_32_N_4_O_4_, 441.2496; found, 441.2505. Anal. found: C, 65.63; H, 7.50; N, 12.71. C_24_H_32_N_4_O_4_ requires C, 65.43; H, 7.32; N, 12.71.

**General procedure for the preparation of bis-(*N*-alkylurea) benzyl esters 2.** A mixture of *O*-succinimidyl carbamate **6** (10.0 mmol) and diisopropylethylamine (1.8 mL, 10.0 mmol) was added to a THF solution (100 mL) of the diamino benzyl ester **4** (2.2 g, 5.0 mmol). The reaction mixture was stirred at 25 °C for 4 h. The insoluble crude solid product was filtered and washed successively with boiling *n*-hexane (100 mL), acetone (100 mL) and THF (100 mL) to afford the title compound.

**2** (*n* = 3). The product was obtained as a white solid (3.0 g, 94%) from *O*-succinimidyl butylcarbamate **6** (*n* = 3) (2.2 g, 10.0 mmol). [α]_D_ = +62.4 (*c* 1.01). mp 221–222 °C. ^1^H NMR (DMSO*-d*_6_): δ 0.83–0.90 (18H, m, CH(C*H*_3_)*_2_* and CH_2_CH_2_C*H_3_*), 1.22–1.36 (8H, m, aliphatic *H*), 1.92–1.94 (2H, m, CHC*H*(CH_3_)_2_), 2.99 (4H, q, *J* = 6, NHC*H_2_*CH_2_), 4.18 (2H, dd, *J* = 8.7 and 6.6, NHC*H*CH), 5.34 (2H, s, PhC*H_2_*), 6.05 (2H, d, *J* = 4.2, urea N*H*), 6.09 (2H, t, *J* = 5.6, urea N*H*), 7.35–7.47 (5H, m, Ar*H*), 7.99 (2H, s, Ar*H*), 8.28 (1H, s, Ar*H*), 10.30 (2H, s, CON*H*Ar). ^13^C NMR (DMSO-*d*_6_): δ 13.7, 17.9, 19.3, 19.5, 31.2, 32.1, 38.9, 58.7, 66.4, 114.4, 114.8, 128.2, 128.4, 128.6, 130.3, 136.0, 139.6, 157.9, 165.4, 171.8. MS (FAB) 639 (M + H^+^, 12%). HRMS (LSIMS): calcd for C_34_H_50_N_6_O_6_, 639.3865; found, 639.3854. Anal. found: C, 63.75; H, 7.95; N, 13.08. C_34_H_50_N_6_O_6_ requires C, 63.93; H, 7.89; N, 13.15.

**2** (*n* = 4). The compound was obtained as a white solid (3.1 g, 94%) from *O*-succinimidyl pentylcarbamate **6** (*n* = 4) (2.3 g, 10.0 mmol). [α]_D_ = +57.9 (*c* 1.08). mp 224–225 °C. ^1^H NMR (DMSO*-d*_6_): δ 0.82–0.90 (18 H, m, CH(C*H*_3_)*_2_* and CH_2_CH_2_C*H_3_*), 1.20–1.39 (12 H, m, aliphatic *H*), 1.91–1.95 (2H, m, CHC*H*(CH_3_)_2_), 2.97 (4H, q, *J* = 6, NHC*H_2_*CH_2_), 4.16 (2H, dd, *J* = 9.0 and 6.6, NHC*H*CH), 5.34 (2H, s, PhC*H_2_*), 6.05 (2H, d, *J* = 4.5, urea N*H*), 6.08 (2H, t, *J* = 5.6, urea N*H*), 7.38–7.48 (5H, m, Ar*H*), 7.98 (2H, s, Ar*H*), 8.26 (1H, s, Ar*H*), 10.30 (2 H, s, CON*H*Ar). ^13^C NMR (DMSO-*d*_6_): δ 13.9, 17.9, 19.3, 21.9, 28.6, 29.6, 31.2, 39.2, 58.7, 66.4, 114.4, 114.8, 128.1, 128.2, 128.6, 130.3, 136.0, 139.6, 157.9, 165.3, 171.8. MS (FAB) 667 (M + H^+^, 10%). HRMS (LSIMS): calcd for C_36_H_54_N_6_O_6_, 667.4178; found, 667.4185. Anal. found: C, 64.77; H, 8.20; N, 12.53. C_36_H_54_N_6_O_6_ requires C, 64.84; H, 8.16; N, 12.60.

**2** (*n* = 5). The product was obtained as a white solid (3.2 g, 91%) from *O*-succinimidyl hexylcarbamate **6** (*n* = 5) (2.4 g, 10.0 mmol). [α]_D_ = +63.6 (*c* 0.99). mp 225–226 °C. ^1^H NMR (DMSO*-d*_6_): δ 0.79–0.89 (18H, m, CH(C*H*_3_)*_2_* and CH_2_CH_2_C*H_3_*), 1.21–1.35 (16H, m, aliphatic *H*), 1.89–1.96 (2H, m, CHC*H*(CH_3_)_2_), 2.95 (4H, q, *J* = 6, NHC*H_2_*CH_2_), 4.17 (2H, dd, *J* = 8.4 and 6.6, NHC*H*CH), 5.31 (2H, s, PhC*H_2_*), 6.00 (2H, d, *J* = 9, urea N*H*), 6.04 (2H, t, *J* = 5.6, urea N*H*), 7.32–7.44 (5H, m, Ar*H*), 7.96 (2H, s, Ar*H*), 8.24 (1H, s, Ar*H*), 10.20 (2H, s, CON*H*Ar). ^13^C NMR (DMSO-*d*_6_): δ 13.9, 17.9, 19.3, 22.1, 26.1, 29.9, 31.0, 31.2, 39, 58.7, 66.4, 114.4, 114.8, 128.1, 128.4, 128.5, 130.3, 136.0, 139.6, 157.9, 165.4, 171.8. MS (FAB) 695 (M + H^+^, 12%). HRMS (LSIMS): calcd for C_38_H_58_N_6_O_6_, 695.4491; found, 695.4501. Anal. found: C, 65.26; H, 8.43; N, 11.81. C_38_H_58_N_6_O_6_ requires C, 65.68; H, 8.41; N, 12.09.

**2** (*n* = 6). The product was obtained as a white solid (3.4 g, 94%) from *O*-succinimidyl heptylcarbamate **6** (*n* = 6) (2.6 g, 10.0 mmol). [α]_D_ = +59.7 (*c* 1.05). mp 227–228 °C. ^1^H NMR (DMSO*-d*_6_): δ 0.81–0.91 (18H, m, CH(C*H*_3_)*_2_* and CH_2_CH_2_C*H_3_*), 1.23–1.37 (20H, m, aliphatic *H*), 1.90–1.97 (2H, m, CHC*H*(CH_3_)_2_), 2.98 (4H, q, *J* = 6, NHC*H_2_*CH_2_), 4.18 (2H, dd, *J* = 9.0 and 6.6, NHC*H*CH), 5.34 (2H, s, PhC*H_2_*), 6.04 (2H, d, *J* = 9.3, urea N*H*), 6.07 (2H, t, *J* = 5.7, urea N*H*), 7.35–7.48 (5H, m, Ar*H*), 7.99 (2H, s, Ar*H*), 8.27 (1H, s, Ar*H*), 10.26 (2H, s, CON*H*Ar). ^13^C NMR (DMSO-*d*_6_): δ 13.9, 17.9, 19.3, 22.0, 26.3, 28.4, 30.0, 31.2, 31.3, 39, 58.7, 66.3, 114.4, 114.8, 128.1, 128.4, 128.5, 130.3, 136.0, 139.6, 157.9, 165.3, 171.8. MS (FAB) 723 (M + H^+^, 5%). HRMS (LSIMS): calcd for C_40_H_62_N_6_O_6_, 723.4804; found, 723.4819. Anal. found: C, 66.42; H, 8.68; N, 11.63. C_40_H_62_N_6_O_6_ requires C, 66.45; H, 8.64; N, 11.62.

**2** (*n* = 9). The product was obtained as a white solid (3.7 g, 92%) from *O*-succinimidyl decylcarbamate **6** (*n* = 9) (3.0 g, 10.0 mmol). [α]_D_ = +53.9 (*c* 1.00). mp 230–231 °C. ^1^H NMR (DMSO*-d*_6_): δ 0.82–0.91 (18H, m, CH(C*H*_3_)*_2_* and CH_2_CH_2_C*H_3_*), 1.22–1.35 (32H, m, aliphatic *H*), 1.91–1.97 (2H, m, CHC*H*(CH_3_)_2_), 2.98 (4 H, q, *J* = 6, NHC*H_2_*CH_2_), 4.18 (2H, dd, *J* = 9.0 and 6.6, NHC*H*CH), 5.34 (2H, s, PhC*H_2_*), 6.03 (2H, d, *J* = 8.7, urea N*H*), 6.06 (2H, t, *J* = 3.8, urea N*H*), 7.35–7.47 (5H, m, Ar*H*), 7.99 (2H, s, Ar*H*), 8.26 (1H, s, Ar*H*), 10.23 (2 H, s, CON*H*Ar). ^13^C NMR (DMSO-*d*_6_): δ 13.9, 17.9, 19.3, 22.1, 26.4, 28.7, 28.8, 28.9, 29.0, 30.0, 31.3, 39, 58.7, 66.3, 114.4, 114.8, 128.1, 128.2, 128.5, 130.3, 136.0, 139.5, 157.9, 165.3, 171.8. MS (FAB) 808 (M + H^+^, 5%). HRMS (LSIMS): calcd for C_46_H_74_N_6_O_6_, 807.5743; found, 807.5730. Anal. found: C, 67.97; H, 9.24; N, 10.39. C_46_H_74_N_6_O_6_ requires C, 68.45; H, 9.24; N, 10.41.

**2** (*n* = 10). The product was obtained as a white solid (3.8 g, 91%) from *O*-succinimidyl undecylcarbamate **6** (*n* = 10) (3.1 g, 10.0 mmol). [α]_D_ = +52.3 (*c* 1.02). mp 232–233 °C. ^1^H NMR (DMSO*-d*_6_): δ 0.81–0.90 (18H, m, CH(C*H*_3_)*_2_* and CH_2_CH_2_C*H_3_*), 1.22–1.34 (36H, m, aliphatic *H*), 1.91–1.97 (2H, m, CHC*H*(CH_3_)_2_), 2.97 (4 H, q, *J* = 6, NHC*H_2_*CH_2_), 4.18 (2H, dd, *J* = 8.7 and 6.6, NHC*H*CH), 5.34 (2H, s, PhC*H_2_*), 6.04 (2H, d, *J* = 5.4, urea N*H*), 6.07 (2H, t, *J* = 5.3, urea N*H*), 7.35–7.47 (5H, m, Ar*H*), 7.98 (2H, s, Ar*H*), 8.26 (1H, s, Ar*H*), 10.27 (2H, s, CON*H*Ar). ^13^C NMR (DMSO-*d*_6_): δ 14.0, 18.0, 19.4, 22.2, 26.5, 28.8, 28.9, 29.1, 29.2, 30.1, 30.8, 31.3, 31.4, 39, 58.9, 66.5, 114.6, 114.9, 128.2, 128.3, 128.7, 130.4, 136.1, 139.7, 158.1, 165.5, 171.9. MS (FAB) 836 (M + H^+^, 5%). HRMS (LSIMS): calcd for C_48_H_78_N_6_O_6_, 835.6056; found, 835.6041. Anal. found: C, 68.67; H, 9.45; N, 10.05. C_48_H_78_N_6_O_6_ requires C, 69.03; H, 9.41; N, 10.06.

**2** (*n* = 12). The product was obtained as a white solid (4.0 g, 90%) from *O*-succinimidyl tridecylcarbamate **6** (*n* = 12) (3.4 g, 10.0 mmol). [α]_D_ = +53.9 (*c* 0.99). mp 235–236 °C. ^1^H NMR (DMSO*-d*_6_, 100 °C): δ 0.82–0.90 (18 H, m, CH(C*H*_3_)*_2_* and CH_2_CH_2_C*H_3_*), 1.23–1.35 (44H, m, aliphatic *H*), 1.91–1.98 (2H, m, CHC*H*(CH_3_)_2_), 2.99 (4H, q, *J* = 7, NHC*H_2_*CH_2_), 4.18 (2H, dd, *J* = 9.0 and 6.6, NHC*H*CH), 5.34 (2H, s, PhC*H_2_*), 6.02 (2H, d, *J* = 9.6, urea N*H*), 6.05 (2H, t, *J* = 6.2, urea N*H*), 7.35–7.47 (5H, m, Ar*H*), 7.98 (2H, s, Ar*H*), 8.26 (1H, s, Ar*H*), 10.20 (2H, s, CON*H*Ar). ^13^C NMR (DMSO-*d*_6_, 100 °C): δ 13.0, 17.3, 18.5, 21.3, 25.8, 27.9, 28.1, 28.3, 29.3, 30.4, 30.6, 39, 58.7, 65.7, 114.9, 115.0, 127.2, 127.4, 127.8, 130.0, 135.7, 138.9, 157.4, 164.9, 171.0. MS (FAB) 892 (M + H^+^, 3%). HRMS (LSIMS): calcd for C_52_H_86_N_6_O_6_, 891.6682; found, 891.6691. Anal. found: C, 69.95; H, 9.79; N, 9.47. C_52_H_86_N_6_O_6_ requires C, 70.08; H, 9.73; N, 9.42.

**2** (*n* = 15). The product was obtained as a white solid (4.2 g, 89%) from *O*-succinimidyl hexadecylcarbamate **6** (*n* = 15) (3.8 g, 10.0 mmol). [α]_D_ = +50.5 (*c* 1.05). mp 239–240 °C. ^1^H NMR (DMSO*-d*_6_, 100 °C): δ 0.86 (6H, t, *J* = 3.5, CH_2_CH_2_C*H_3_*), 0.91 (6H, d, *J* = 6.9, CH(C*H*_3_)Me), 0.94 (6H, d, *J* = 6.9, CHMe(C*H*_3_)), 1.26–1.40 (56H, m, aliphatic *H*), 1.90–2.15 (2H, m, CHC*H*(CH_3_)_2_), 2.95–3.06 (4H, m, NHC*H_2_*CH_2_), 4.19 (2H, dd, *J* = 6.3 and 9, NHC*H*CH), 5.36 (2H, s, PhC*H_2_*), 5.86 (2H, d, *J* = 9, urea N*H*), 5.93 (2H, t, *J* = 5.4, urea N*H*), 7.37–7.47 (5H, m, Ar*H*), 7.95 (2H, d, *J* = 2.1, Ar*H*), 8.26 (1H, t, *J* = 2.1, Ar*H*), 9.90 (2H, s, CON*H*Ar). ^13^C NMR (DMSO-*d*_6_, 100 °C): δ 13.0, 17.3, 18.6, 21.3, 25.8, 28.0, 28.2, 28.4, 29.4, 30.5, 30.6, 39, 58.6, 65.7, 114.87, 114.93, 127.3, 127.4, 127.9, 130.0, 135.7, 139.0, 157.5, 164.9, 171.0. MS (FAB) 976 (M + H^+^, 5%). HRMS (LSIMS): calcd for C_58_H_98_N_6_O_6_, 975.7621; found, 975.7626. Anal. found: C, 71.46; H, 9.94; N, 8.35. C_58_H_98_N_6_O_6_ requires C, 71.42; H, 10.13; N, 8.61.

**2** (*n* = 18). The product was obtained as a white solid (4.7 g, 88%) from *O*-succinimidyl nonadecylcarbamate **6** (*n* = 18) (4.3 g, 10.0 mmol). [α]_D_ = +52.5 (*c* 1.01). mp 243–244 °C. ^1^H NMR (DMSO*-d*_6_, 100 °C): δ 0.84–0.93 (18H, m, CH(C*H*_3_)*_2_* and CH_2_CH_2_C*H_3_*), 1.25–1.37 (68H, m, aliphatic *H*), 1.95–1.99 (2H, m, CHC*H*(CH_3_)_2_), 2.95–3.06 (4H, m, NHC*H_2_*CH_2_), 4.19 (2H, dd, *J* = 8.4 and 6.6, NHC*H*CH), 5.35 (2H, s, PhC*H_2_*), 5.92 (2H, d, *J* = 8.7, urea N*H*), 5.98 (2H, t, *J* = 5.6, urea N*H*), 7.35–7.47 (5H, m, Ar*H*), 7.96 (2H, s, Ar*H*), 8.24 (1H, s, Ar*H*), 10.04 (2H, s, CON*H*Ar). ^13^C NMR (DMSO-*d*_6_, 100 °C): δ 13.0, 17.3, 18.6, 21.3, 25.8, 28.0, 28.2, 28.4, 29.4, 30.5, 30.6, 39, 58.6, 65.7, 114.87, 114.93, 127.3, 127.4, 127.9, 130.0, 135.7, 139.0, 157.5, 164.9, 171.0. MS (FAB) 1060 (M + H^+^, 3%). HRMS (LSIMS): calcd for C_64_H_110_N_6_O_6_, 1069.8560; found, 1069.8550. Anal. found: C, 72.53; H, 10.57; N, 7.81. C_64_H_110_N_6_O_6_ requires C, 72.55; H, 10.46; N, 7.93.

**2** (*n* = 20). The product was obtained as a white solid (4.8 g, 86%) from *O*-succinimidyl heneicosylcarbamate **6** (*n* = 20) (4.5 g, 10.0 mmol). [α]_D_ = +53.1 (*c* 1.00). mp 249–250 °C. ^1^H NMR (DMSO*-d*_6_, 100 °C): δ 0.86 (6H, t, *J* = 6.9, CH_2_CH_2_C*H_3_*), 0.92 (6H, d, *J* = 6.9, CH(C*H*_3_)Me), 0.95 (6H, d, *J* = 6.9, CHMe(C*H*_3_)), 1.28–1.43 (76H, m, aliphatic *H*), 1.95–2.12 (2H, m, CHC*H*(CH_3_)_2_), 3.00 (4H, q, *J* = 6, NHC*H_2_*CH_2_), 4.19 (2H, dd, *J* = 8.1 and 6.6, NHC*H*CH), 5.36 (2H, s, PhC*H_2_*), 5.82 (2H, d, *J* = 9, urea N*H*), 5.88 (2H, t, *J* = 5.6, urea N*H*), 7.37–7.47 (5H, m, Ar*H*), 7.94 (2H, d, *J* = 2.1, Ar*H*), 8.19 (1H, t, *J* = 1.8, Ar*H*), 9.80 (2H, s, CON*H*Ar). ^13^C NMR (DMSO-*d*_6_, 100 °C): δ 13.0, 17.3, 18.5, 20.2, 21.3, 25.8, 27.9, 28.1, 28.3, 29.3, 30.4, 30.6, 39, 58.7, 65.7, 114.9, 115.0, 127.2, 127.4, 127.8, 130.0, 135.7, 138.9, 157.5, 164.9, 171.0. MS (FAB) 1116 (M + H^+^, 100%). HRMS (LSIMS): calcd for C_68_H_118_N_6_O_6_, 1115.9186; found, 1115.9192. Anal. found: C, 73.17; H, 10.53; N, 7.45. C_68_H_118_N_6_O_6_ requires C, 73.20; H, 10.66; N, 7.53.

## Supporting Information

General procedure, yields and characterization data of compounds 6 (*n* = 3–20).

File 1 Experimental Part.
