# Supplementary material for: Expanding the gelation properties of valine-based 3,5-diaminobenzoate organogelators with N-alkylurea functionalities
Source: Beilstein J Org Chem. 2010 Oct 26;6:1015–21. doi: 10.3762/bjoc.6.114 (PMC2981810; doi:10.3762/bjoc.6.114)
Supplement: File 1 — Experimental Part. [file Beilstein_J_Org_Chem-06-1015-s001.pdf]

Supporting Information

for

Expanding the gelation properties of valine-based 3,5-diaminobenzoate organogelators with *N*-alkylurea functionalities

Hak-Fun Chow*[1,2,3] and Chin-Ho Cheng[1]

[1]Department of Chemistry, The Chinese University of Hong Kong, Shatin, NT, Hong Kong SAR, [2]The Center of Novel Functional Molecules, The Chinese University of Hong Kong, Shatin, NT, Hong Kong SAR and [3]Institute of Molecular Functional Materials, Areas of Excellence Scheme, University Grants Committee, Hong Kong SAR

Email: Hak-Fun Chow - hfchow@cuhk.edu.hk

* Corresponding author

General procedure, yields and characterization data of compounds 6 (*n* = 3–20).

General procedure for the preparation of *O*-succinimidyl alkylcarbamates 6. A mixture of EtOCOCl (11.5 mL, 0.12 mol) and *N*-methyl morpholine (NMM) (13.2 mL, 0.12 mol) was added to a THF solution (250 mL) of the carboxylic acid **5** (0.10 mol). The reaction mixture was stirred at −20 °C for 30 min. An aqueous NaN_3 solution (16.3 g in 100 mL water, 0.25 mol) was then added and the mixture stirred under a nitrogen atmosphere at −5 °C for 15 min. The reaction mixture was extracted with EtOAc (100 mL × 3) and the combined organic layers were washed with saturated NaCl solution (100 mL), dried ($MgSO_4$), filtered and concentrated in vacuo. The crude reaction product was then heated in toluene (100 mL) at 65 °C for 30 min until gas evolution ceased. Solid *N*-hydroxysuccinimide (11.5 g, 0.10 mol) and pyridine (8.1 mL, 0.10 mol) were added successively and the reaction mixture was stirred for 2 h at room temperature. The product was precipitated in hexane/EtOAc (2/1), filtered and washed with diethyl ether (100 mL). Compounds **6** were obtained as crystalline solids after flash chromatography (hexane/EtOAc = 1/1).

***O*-Succinimidyl butylcarbamate** (**6**, *n* = 3). Starting from pentanoic acid (10.2 g, 0.10 mol), the product was obtained as a white solid (18.3 g, 85%). R_f 0.40 (hexane/EtOAc = 1/1). mp 71–72 °C. ^{1}H NMR ($CDCl_3$): δ 0.93 (3 H, t, *J* = 6.0, CH_2CH_3), 1.33–1.59 (4 H, m, aliphatic *H*), 2.82 (4 H, s, $COCH_2$), 3.24 (2 H, q, *J* = 7.0, $NHCH_2CH_2$), 5.36 (1 H, s, CON*H*). ^{13}C NMR ($CDCl_3$): δ 13.6, 19.7, 25.4, 31.4, 41.7, 151.6, 170.6. MS (FAB) 215 (M + H^+, 45%). HRMS (L SIMS): calcd for $C_9H_{14}N_2O_4$, 215.1026; found, 215.1030. Anal. found: C, 50.55; H, 6.64; N, 13.01. $C_9H_{14}N_2O_4$ requires C, 50.46; H, 6.59; N, 13.07.

***O*-Succinimidyl pentylcarbamate** (**6**, *n* = 4). Starting from hexanoic acid (11.6 g, 0.10 mol), the product was obtained as a white solid (19.3 g, 84%). R_f 0.42 (hexane/EtOAc = 1/1). mp 74–

75 °C. ^{1}H NMR ($CDCl_3$): δ 0.90 (3 H, t, *J* = 7.5, CH_2CH_3), 1.30–1.59 (6 H, m, aliphatic *H*), 2.82 (4 H, s, $COCH_2$), 3.25 (2 H, q, *J* = 7.0, $NHCH_2CH_2$), 5.24 (1 H, s, CON*H*). ^{13}C NMR ($CDCl_3$): δ 13.8, 22.1, 25.4, 28.6, 28.9, 41.9, 151.5, 170.6. MS (FAB) 229 (M + H^+, 25%). HRMS (L SIMS): calcd for $C_{10}H_{16}N_2O_4$, 229.1183; found, 229.1175. Anal. found: C, 52.49; H, 7.05; N, 12.16. $C_{10}H_{16}N_2O_4$ requires C, 52.62; H, 7.07; N, 12.27.

***O*-Succinimidyl hexylcarbamate** (**6**, *n* = 5). Starting from heptanoic acid (13.0 g, 0.10 mol), the product was obtained as a white solid (20.4 g, 84%). R_f 0.43 (hexane/EtOAc = 1/1). mp 76–77 °C. ^{1}H NMR ($CDCl_3$): δ 0.89 (3 H, t, *J* = 6.0, CH_2CH_3), 1.20–1.50 (8 H, m, aliphatic *H*), 2.81 (4 H, s, $COCH_2$), 3.23 (2 H, q, *J* = 7.0, $NHCH_2CH_2$), 5.68 (1 H, s, CON*H*). ^{13}C NMR ($CDCl_3$): δ 14.1, 22.6, 25.6, 26.4, 29.5, 31.4, 42.2, 151.5, 170.2. MS (FAB) 243 (M + H^+, 25%). HRMS (L SIMS): calcd for $C_{11}H_{18}N_2O_4$, 243.1339; found, 243.1348. Anal. found: C, 54.50; H, 7.53; N, 11.48. $C_{11}H_{18}N_2O_4$ requires C, 54.53; H, 7.49 ; N, 11.56.

***O*-Succinimidyl heptylcarbamate** (**6**, *n* = 6). Starting from octanoic acid (14.4 g, 0.10 mol), the product was obtained as a white solid (21.6 g, 85%). R_f 0.43 (hexane/EtOAc = 1/1). mp 78–79 °C. ^{1}H NMR ($CDCl_3$): δ 0.89 (3 H, t, *J* = 6.0, CH_2CH_3), 1.28 (8 H, m, aliphatic *H*), 1.53 (2 H, q, *J* = 7.0, aliphatic *H*), 2.81 (4 H, s, $COCH_2$), 3.22 (2 H, q, *J* = 7.0, $NHCH_2CH_2$), 5.63 (1 H, s, CON*H*). ^{13}C NMR ($CDCl_3$): δ 14.0, 22.5, 25.4, 25.5, 26.3, 29.4, 31.3, 42.1, 151.6, 170.6. MS (FAB) 257 (M + H^+, 35%). HRMS (L SIMS): calcd for $C_{12}H_{20}N_2O_4$, 257.1469; found, 257.1507. Anal. found: C, 56.22; H, 8.02; N, 10.75. $C_{12}H_{20}N_2O_4$ requires C, 56.24; H, 7.86; N, 10.92.

***O*-Succinimidyl decylcarbamate** (**6**, *n* = 9). Starting from undecanoic acid (18.6 g, 0.10 mol), the product was obtained as a white solid (23.9 g, 80%). R_f 0.46 (hexane/EtOAc = 1/1). mp 81–

82 °C. ^{1}H NMR ($CDCl_3$): δ 0.88 (3 H, t, *J* = 6.0, CH_2CH_3), 1.26–1.30 (14 H, m, aliphatic *H*), 1.54–1.56 (2 H, m, aliphatic *H*), 2.82 (4 H, s, $COCH_2$), 3.25 (2 H, q, *J* = 7.0, $NHCH_2CH_2$), 5.21 (1 H, s, CON*H*). ^{13}C NMR ($CDCl_3$): δ 14.1, 22.7, 25.4, 26.6, 29.2, 29.3, 29.4, 29.5, 29.6, 31.9, 42.0, 151.5, 170.6. MS (FAB) 299 (M + H^+, 25%). HRMS (L SIMS): calcd for $C_{15}H_{26}N_2O_4$, 299.1965; found, 299.1961. Anal. found: C, 60.46; H, 8.96; N, 9.29. $C_{15}H_{26}N_2O_4$ requires C, 60.38; H, 8.78; N, 9.38.

***O*-Succinimidyl undecylcarbamate** (**6**, *n* = 10). Starting from dodecanoic acid (20.0 g, 0.10 mol), the product was obtained as a white solid (24.4 g, 78%). R_f 0.48 (hexane/EtOAc = 1/1). mp 82–83 °C. ^{1}H NMR ($CDCl_3$): δ 0.82 (3 H, t, *J* = 6.6, CH_2CH_3), 1.20–1.67 (18 H, m, aliphatic *H*), 2.82 (4 H, s, $COCH_2$), 3.21 (2 H, q, *J* = 6.4, $NHCH_2CH_2$), 6.26 (1 H, s, CON*H*). ^{13}C NMR ($CDCl_3$): δ 14.2, 22.8, 25.6, 26.7, 29.3, 29.45, 29.55, 29.6, 29.68, 29.74, 32.0, 42.2, 151.5, 170.3. MS (FAB) 313 (M + H^+, 33%). HRMS (L SIMS): calcd for $C_{16}H_{28}N_2O_4$, 313.2122; found, 313.2106. Anal. found: C, 61.39; H, 9.17; N, 8.91. $C_{16}H_{28}N_2O_4$ requires C, 61.51; H, 9.03; N, 8.96.

***O*-Succinimidyl tridecylcarbamate** (**6**, *n* = 12). Starting from tetradecanoic acid (22.8 g, 0.10 mol), the product was obtained as a white solid (26.2 g, 77%). R_f 0.48 (hexane/EtOAc = 1/1).mp 85–86 °C. ^{1}H NMR ($CDCl_3$): δ 0.88 (3 H, t, *J* = 6.6, CH_2CH_3), 1.25–1.65 (22 H, m, aliphatic *H*), 2.82 (4 H, s, $COCH_2$), 3.21 (2 H, q, *J* = 6.6, $NHCH_2CH_2$), 5.15 (1 H, s, CON*H*). ^{13}C NMR ($CDCl_3$): δ 14.2, 22.8, 25.6, 26.7, 29.3, 29.5, 29.55, 29.60, 29.68, 29.74, 32.0, 42.2, 151.5, 170.3. MS (FAB) 341 (M + H^+, 28%). HRMS (L SIMS): calcd for $C_{18}H_{32}N_2O_4$, 341.2435; found, 341.2429. Anal. found: C, 63.50; H, 9.56; N, 8.02. $C_{18}H_{32}N_2O_4$ requires C, 63.50; H, 9.47; N, 8.22.

***O*-Succinimidyl hexadecylcarbamate** (**6**, *n* = 15). Starting from heptadecanoic acid (27.0 g, 0.10 mol), the product was obtained as a white solid (30.3 g, 79%). R_f 0.49 (hexane/EtOAc = 1/1). mp 88–89 °C. ^{1}H NMR ($CDCl_3$): δ 0.88 (3 H, t, *J* = 7.2, CH_2CH_3), 1.19–1.68 (28 H, m, aliphatic *H*), 2.82 (4 H, s, $COCH_2$), 3.24 (2 H, q, *J* = 6.7, $NHCH_2CH_2$), 5.30 (1 H, s, CON*H*). ^{13}C NMR ($CDCl_3$): δ 14.2, 22.8, 25.6, 26.7, 29.3, 29.5, 29.6, 29.7, 29.8, 32.0, 42.2, 151.5, 170.2. MS (FAB) 383 (M + H^+, 12%). HRMS (L SIMS): calcd for $C_{21}H_{38}N_2O_4$, 383.2904; found, 383.2914. Anal. found: C, 65.91; H, 10.23; N, 7.19. $C_{21}H_{38}N_2O_4$ requires C, 65.94; H, 10.01; N, 7.32.

***O*-Succinimidyl nonadecylcarbamate** (**6**, *n* = 18). Starting from eicosanoic acid (31.2 g, 0.10 mol), the product was obtained as a white solid (32.3 g, 76%). R_f 0.50 (hexane/EtOAc = 1/1). mp 90–92 °C. ^{1}H NMR ($CDCl_3$): δ 0.88 (3 H, t, *J* = 6.6, CH_2CH_3), 1.20–1.58 (34 H, m, aliphatic *H*), 2.82 (4 H, s, $COCH_2$), 3.25 (2 H, q, *J* = 7.8, $NHCH_2CH_2$), 5.23 (1 H, s, CON*H*). ^{13}C NMR ($CDCl_3$): δ 14.3, 22.8, 25.6, 26.7, 29.3, 29.5, 29.6, 29.7, 29.8, 32.1, 42.2, 151.5, 170.1. MS (FAB) 425 (M + H^+, 18%). HRMS (L SIMS): calcd for $C_{24}H_{44}N_2O_4$, 425.3374; found, 425.3358. Anal. found: C, 67.74; H, 10.60; N, 6.44. $C_{24}H_{44}N_2O_4$ requires C, 67.89; H, 10.44; N, 6.59.

***O*-Succinimidyl heneicosylcarbamate** (**6**, *n* = 20). Starting from docosanoic acid (33.0 g, 0.10 mol), the product was obtained as a white solid (34.4 g, 76%). R_f 0.50 (hexane/EtOAc = 1/1). mp 92–94 °C. ^{1}H NMR ($CDCl_3$): δ 0.86 (3 H, t, CH_2CH_3), 1.18–1.57 (38 H, m, aliphatic *H*), 2.82 (4 H, d, $COCH_2$), 3.24 (2 H, q, *J* = 6.7, $NHCH_2CH_2$), 5.23 (1 H, s, N*H*). ^{13}C NMR ($CDCl_3$): δ 14.3, 22.8, 25.6, 26.7, 29.3, 29.5, 29.6, 29.7, 29.8, 32.1, 42.3, 151.5, 170.1. MS

(FAB) 453 (M + H$^+$, 11%). HRMS (L SIMS): calcd for $C_{26}H_{48}N_2O_4$, 453.3698; found, 453.3689. Anal. found: C, 68.77; H, 10.87; N, 6.03. $C_{26}H_{48}N_2O_4$ requires C, 68.99; H, 10.69; N, 6.19.
